# Meta-analysis of risk factors for postoperative lower extremity deep vein thrombosis in patients with gynecologic malignancies

**DOI:** 10.3389/fsurg.2025.1406425

**Published:** 2025-01-23

**Authors:** Danzhu Gong, Qi Wang

**Affiliations:** Department of Gynecology, Linhai Hospital of Traditional Chinese Medicine, Linhai, Zhejiang, China

**Keywords:** postoperative gynecologic malignancy, lower extremity deep vein thrombosis, risk factors, meta-analysis, prophylaxis, interventions

## Abstract

**Introduction:**

This study aims to elucidate the risk factors associated with postoperative lower extremity deep vein thrombosis (DVT) in patients afflicted with gynecologic malignancies.

**Methods:**

A comprehensive search was conducted across CNKI, CQVIP, Wanfang, and PubMed databases for literature published from January 1, 2024, concerning the risk factors for postoperative DVT in patients with gynecologic cancers. Two researchers independently and objectively screened, extracted, and evaluated the pertinent data. Subsequently, the extracted data were subjected to meta-analysis using STATA software.

**Results:**

A total of twelve studies fulfilling the inclusion criteria were analyzed, encompassing 2,553 cases. The meta-analysis revealed that significant risk factors for postoperative lower extremity DVT in patients with gynecologic malignancies include age [Odds Ratio (OR) = 1.35, 95% Confidence Interval (CI) (1.19, 1.54), *P* < 0.001], Body Mass Index (BMI) [OR = 1.67, 95% CI (1.05, 2.66), *P* < 0.001], plasma D-dimer levels [OR = 3.39, 95% CI (1.24, 9.24), *P* < 0.001], duration of surgery [OR = 2.24, 95% CI (1.24, 4.01), *P* < 0.001], and duration of postoperative bed rest [OR = 2.14, 95% CI (1.56, 2.94), *P* < 0.001].

**Discussion:**

The study identifies multiple risk factors influencing the incidence of postoperative lower extremity DVT in patients with gynecologic malignancies. Notably, age, BMI, plasma D-dimer levels, surgical duration, and postoperative bed rest emerge as significant predictors. These findings underscore the necessity for targeted prophylaxis and therapeutic interventions in the clinical management of such patients.

## Introduction

Deep Vein Thrombosis (DVT) constitutes a pathological condition wherein blood abnormally coagulates within the veins, leading to venous blockage and impaired venous return. The onset of this condition is marked by four principal manifestations: limb swelling, pain, elevated local skin temperature, and superficial vein varicosity. Such a condition may be precipitated by factors including trauma, surgery, neoplasms, and parturition (1), primarily affecting the iliac-femoral and femoral-carotid veins in the lower extremities. Postoperative DVT, typically manifesting within one month following surgical intervention, is precipitated by the surgical and traumatic stimuli. Research grounded in evidence-based medicine indicates that the incidence of postoperative DVT ranges between 42% and 57% in the absence of postoperative thromboprophylaxis. Advancements in surgical techniques, an enhanced understanding of DVT, and improvements in rehabilitative medical care have contributed to a gradual decline in the incidence of postoperative DVT. However, as a grave postoperative complication, DVT not only escalates treatment costs and hinders recovery but also poses the risk of pulmonary embolism—a dire and potentially fatal consequence—underscoring the necessity of its emphasis in clinical practice (2). Investigations have elucidated that DVT, as a prevalent postoperative complication among patients with gynecological surgery due to malignancies (3), significantly impedes the therapeutic process, prolongs hospitalization, diminishes life quality, and may precipitate pulmonary embolism, thus severely jeopardizing patient safety (4). Consequently, the prevention and mitigation of DVT's development in clinical settings are of paramount importance. This study endeavors to meticulously review and analyze literature from both domestic and international sources to analyze risk factors associated with DVT following gynecological surgery, so as to identify and assess the risk factors for DVT occurrence in gynecological postoperative patients, which provides a basis for prevention and control strategies.

## Information and methods

### Criteria for inclusion of literature

(1) The study type of included literature was either cohort or case-control studies; (2) the study participants were patients who had undergone gynecological surgery due to malignancies and subsequently developed deep vein thrombosis (DVT) in the lower extremities; (3) patients who experienced DVT in the lower extremities following gynecological surgery, with imaging diagnoses; (4) multivariate logistic regression analysis was used to identify risk factors for postoperative bowel obstruction in patients undergoing gynecological surgery due to malignancies, providing odds ratios (ORs) and 95% confidence intervals (CIs) for these risk factors, or allowing for the transformation and application of the data.

### Criteria for exclusion of literature

Excluded from this analysis are types of literature including reviews, literature analyses, experiential summaries, theoretical discussions, systematic assessments, clinical observations, animal studies, replicated studies, clinical randomized controlled trials lacking study endpoints, studies with incomplete data, those that fail to provide access to the original literature, cross-sectional analyses, research unrelated to venous thrombosis of the lower extremities, and studies of patients undergoing gynecological surgery but not due to malignancies.

### Literature search strategy

The literature search was conducted through electronic databases such as CNKI, CQVIP, Wanfang, and PubMed using both Chinese and English keywords. The Chinese keywords included terms related to “lower limbs deep venous thrombosis, deep venous thrombosis, post-surgery gynecologic malignant tumors, risk factors,” whereas the English keywords comprised “lower limbs, thrombus, deep venous, thrombosis, DVT, After Surgery; Postsurgical.” The systematical searches were conducted from database inception to January 1, 2024.

### Data extraction and quality assessment

Two researchers independently and impartially conducted the literature search, selection, and data extraction based on predetermined inclusion and exclusion criteria, ensuring the integrity of the original data by designing a data extraction template to facilitate pre-extraction processes. In instances of discordance, a third researcher or a dedicated evidence-based medicine team within the hospital was consulted to verify and reconcile the data. The quality of the included cohort or case-control studies was independently evaluated by the two researchers using the Newcastle-Ottawa Scale (NOS). Discrepancies in the literature quality assessment were resolved through consultation with an in-house evidence-based panel. The NOS framework, designed to assess the quality of non-randomized studies in meta-analyses, includes two segments for evaluating cohort and case-control studies. It comprises three domains: selection of the study groups, comparability of the groups, and ascertainment of either the exposure or outcome, culminating in a comprehensive score out of nine points.

### Statistical methods

Statistical analyses were executed employing RevMan 5.4.1 software. The heterogeneity of study outcomes was assessed using the Chi-squared (*χ*^2^) test and the *I*^2^ statistic. Studies were deemed homogeneous if *P* > 0.1 and *I*^2^ ≤ 50%, warranting the use of a fixed-effects model; conversely, if *P* ≤ 0.1 and *I*^2^ > 50%, indicating heterogeneity, a random-effects model was applied. Sensitivity analyses were conducted to explore the sources of significant heterogeneity. The outcomes of this research, encompassing both continuous and dichotomous variables, were analyzed. Continuous outcomes were evaluated using the mean difference (MD) and standardized mean difference (SMD), while dichotomous outcomes were assessed through odds ratios (OR), with all results accompanied by 95% confidence intervals (CIs). Statistical significance for continuous variables was determined by whether the combined effect size deviated from zero, indicating an association with the disease occurrence. Funnel plots were employed to investigate publication bias for study factors where ten or more studies were included in the analysis.

## Results

### Literature screening outcomes

The search across CNKI, VIP, WanFang, and PubMed databases yielded 356 articles. Upon initial screening, 82 duplicates were excluded. A further 216 articles were deemed irrelevant after reviewing titles and abstracts due to their focus on literature reviews, animal experiments, theoretical discussions, and non-pertinent research, leaving 58 articles for detailed examination. After thorough review, 46 articles were excluded, culminating in the inclusion of 12 high-quality, representative articles for analysis.

Table 1 Basic information and quality scores of the included literature.

**Table 1:** Characteristics and quality assessment of included studies.

Study	DVT/Non-DVT	Thrombus type	Indicator
Chen et al. (5)	80/80	LEDVT	Age, D-dimer
Lv et al. (6)	40/80	LEDVT	Age, BMI, duration of surgery, malignancy, and postoperative bedtime
Yang and He (7)	25/223	LEDVT	Age, BMI, postoperative bed rest
Zhou and Li (8)	35/337	LEDVT	Age, malignancy, D-dimer, duration of surgery, and postoperative bed rest
Han (9)	53/89	LEDVT	Age, BMI, duration of surgery, postoperative bedtime
Ren and Guo (10)	23/272	LEDVT	Age, BMI, Tumor Properties, and D-Dimerization
Zhang and Li (11)	6/54	LEDVT	Age and postoperative bedtime
Qiu (12)	26/249	LEDVT	Age, malignancy, D-dimer, BMI, duration of surgery, and postoperative bed rest
Yang (13)	103/103	LEDVT	Age, BMI, duration of surgery, D-dimer
Li et al. (14)	30/309	LEDVT	Age, malignancy, D-dimer, duration of surgery, and postoperative bed rest
Li (15)	45/45	LEDVT	Age, BMI, malignant tumor, postoperative bed rest
Cui et al. (16)	35/211	LEDVT	Age

LEDVT, lower extremity deep vein thrombosis; BMI, body mass index.

### Meta-analysis results

The meta-analysis focused on six study factors to systematically assess the risk factors associated with postoperative deep vein thrombosis (DVT) in patients with gynecological malignancies. The analyzed outcome indicators included age, body mass index (BMI), D-dimer levels, operation duration, the presence of malignant tumors, and postoperative bed rest duration.

### Age

**Figure 1:**
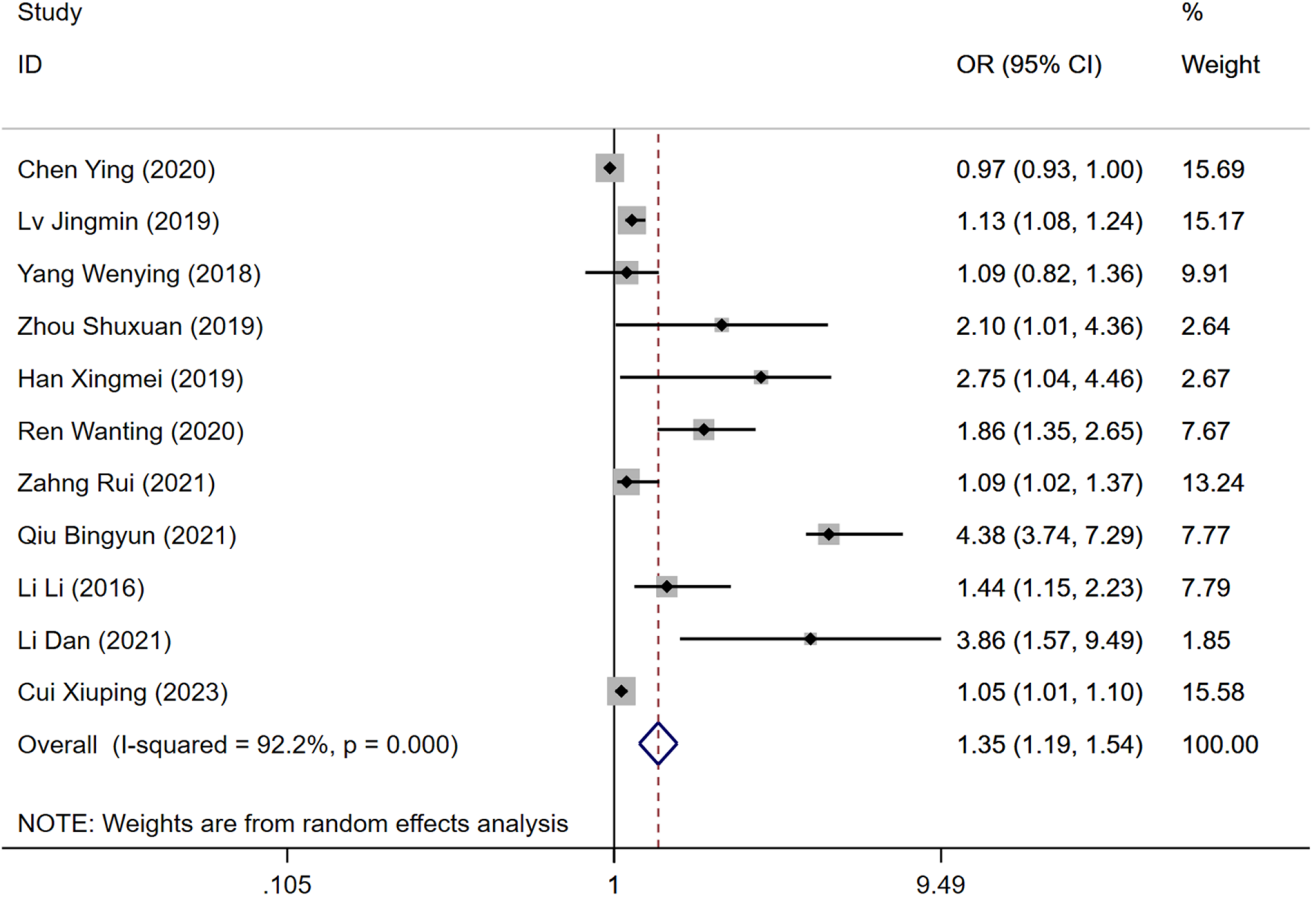
Forest plot of the relationship between age and postoperative DVT in patients with gynecologic malignancies.

Out of the included studies, 12 reported age as a risk factor for DVT development post-gynecological surgery. The heterogeneity test indicated significant heterogeneity (*P* = 0.000, *I*^2^ = 92.2%), necessitating the use of a random-effects model for analysis. The findings demonstrated that age significantly influences the risk of DVT, with the DVT group showing a higher risk compared to the control group [Odds Ratio [OR] = 1.35, 95% Confidence Interval [CI] [1.19, 1.54], *P* = 0.000], as illustrated in Figure 1, which is a forest plot depicting the relationship between age and postoperative DVT in patients with gynecologic malignancies.

### BMI

**Figure 2:**
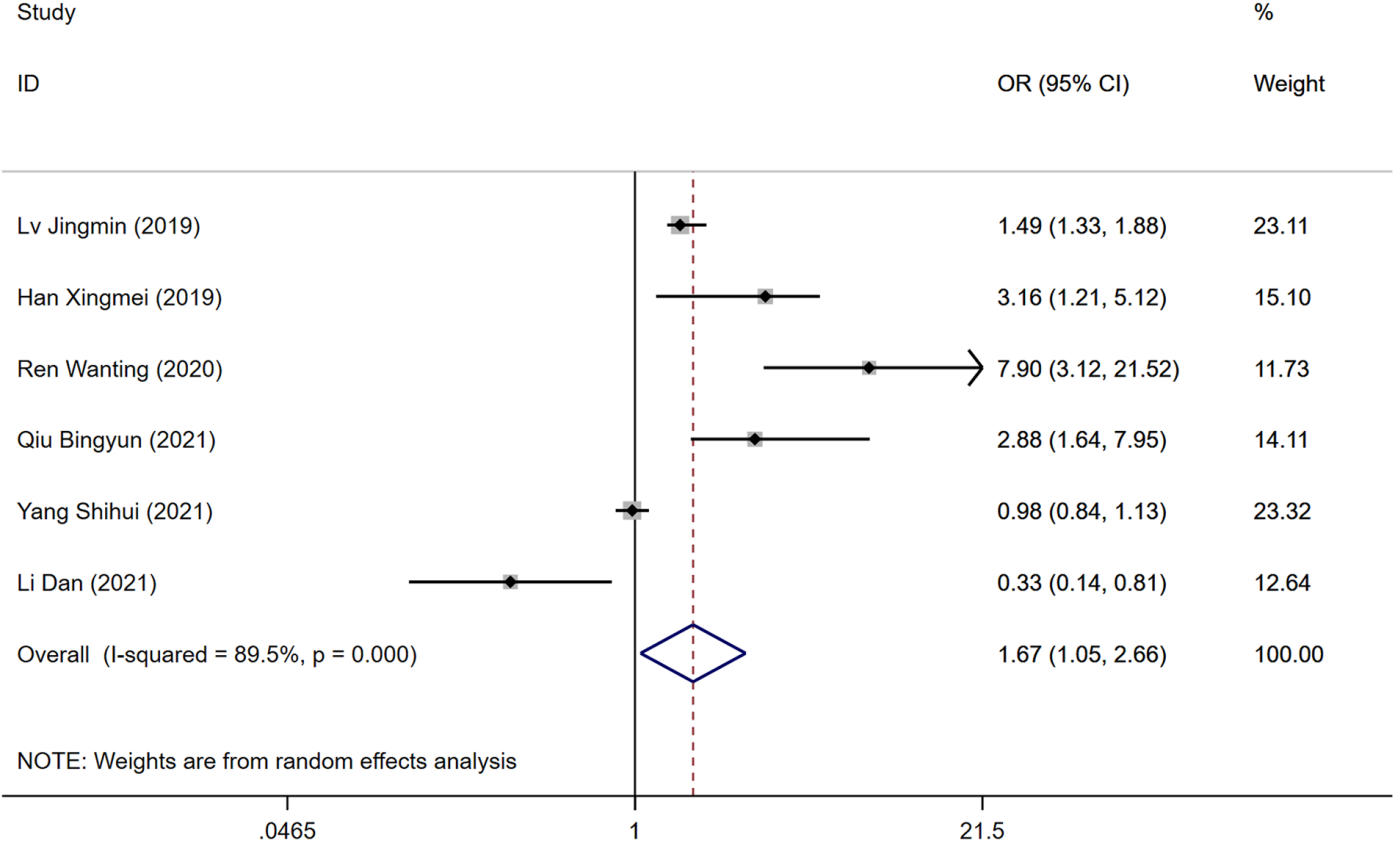
Forest plot of the relationship between BMI and postoperative DVT in patients with gynecologic malignancies.

Six studies identified BMI as a risk factor for DVT development following surgery for gynecological malignancies. Significant heterogeneity was observed (*P* = 0.000, *I*^2^ = 89.5%), analyzed through a random-effects model. The analysis indicated a statistically significant difference in DVT risk between the groups, with a higher risk in the DVT group compared to controls [OR = 1.67, 95% CI (1.05, 2.66), *P* = 0.000], as shown in Figure 2.

### D-dimer

**Figure 3:**
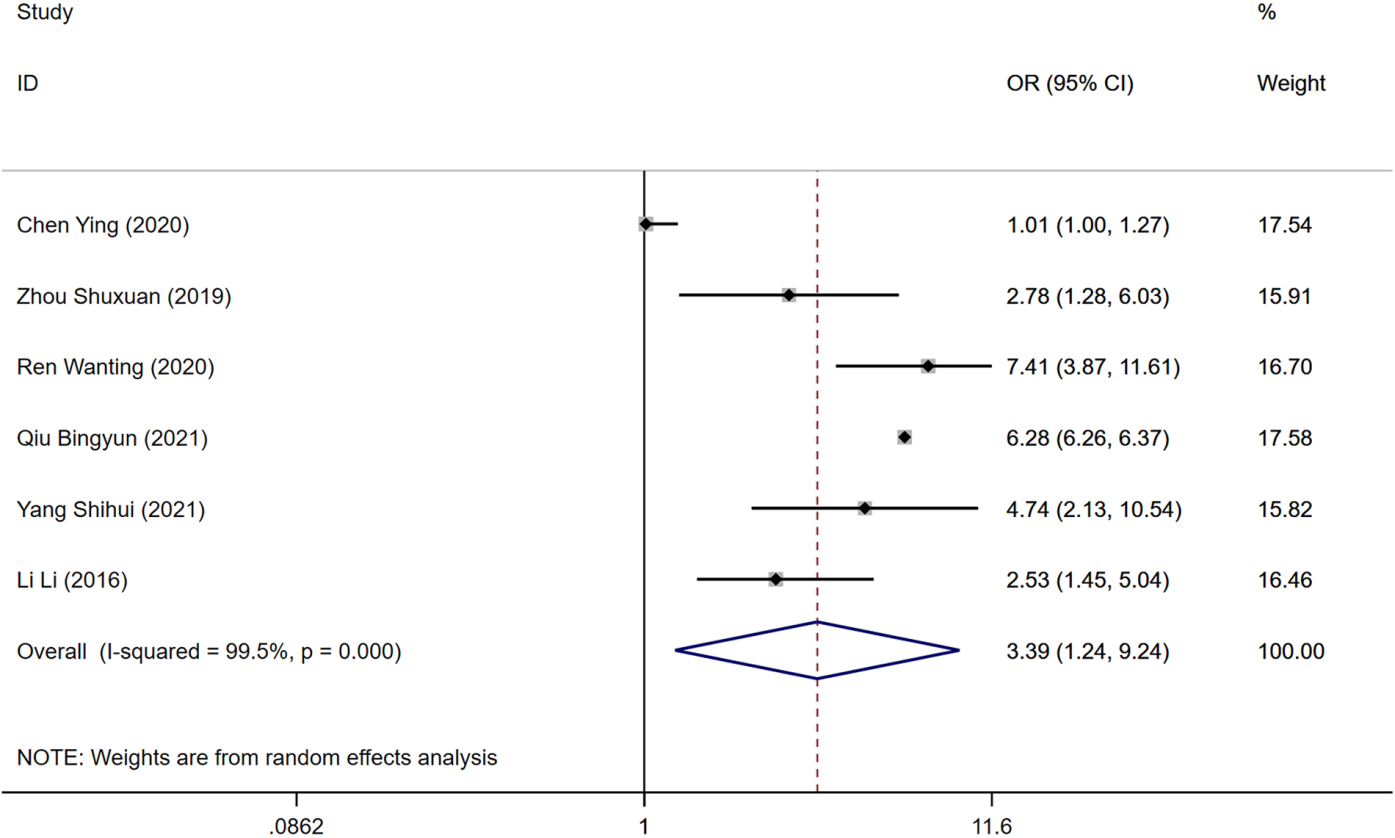
Forest plot of the relationship between D-dimer and postoperative DVT in patients with gynecologic malignancies.

Among the reviewed literature, six studies identified D-dimer levels as a risk factor for the development of DVT following gynecologic malignancy surgeries. Significant heterogeneity was observed in the results (*P* = 0.000, *I*^2^ = 89.5%), prompting analysis through a random-effects model. The elevated levels of D-dimer were significantly associated with an increased risk of DVT in the postoperative period when compared to the control group [Odds Ratio [OR] = 3.39, 95% Confidence Interval [CI] [1.24, 9.24], *P* = 0.000], as depicted in Figure 3.

### Malignant tumors

**Figure 4:**
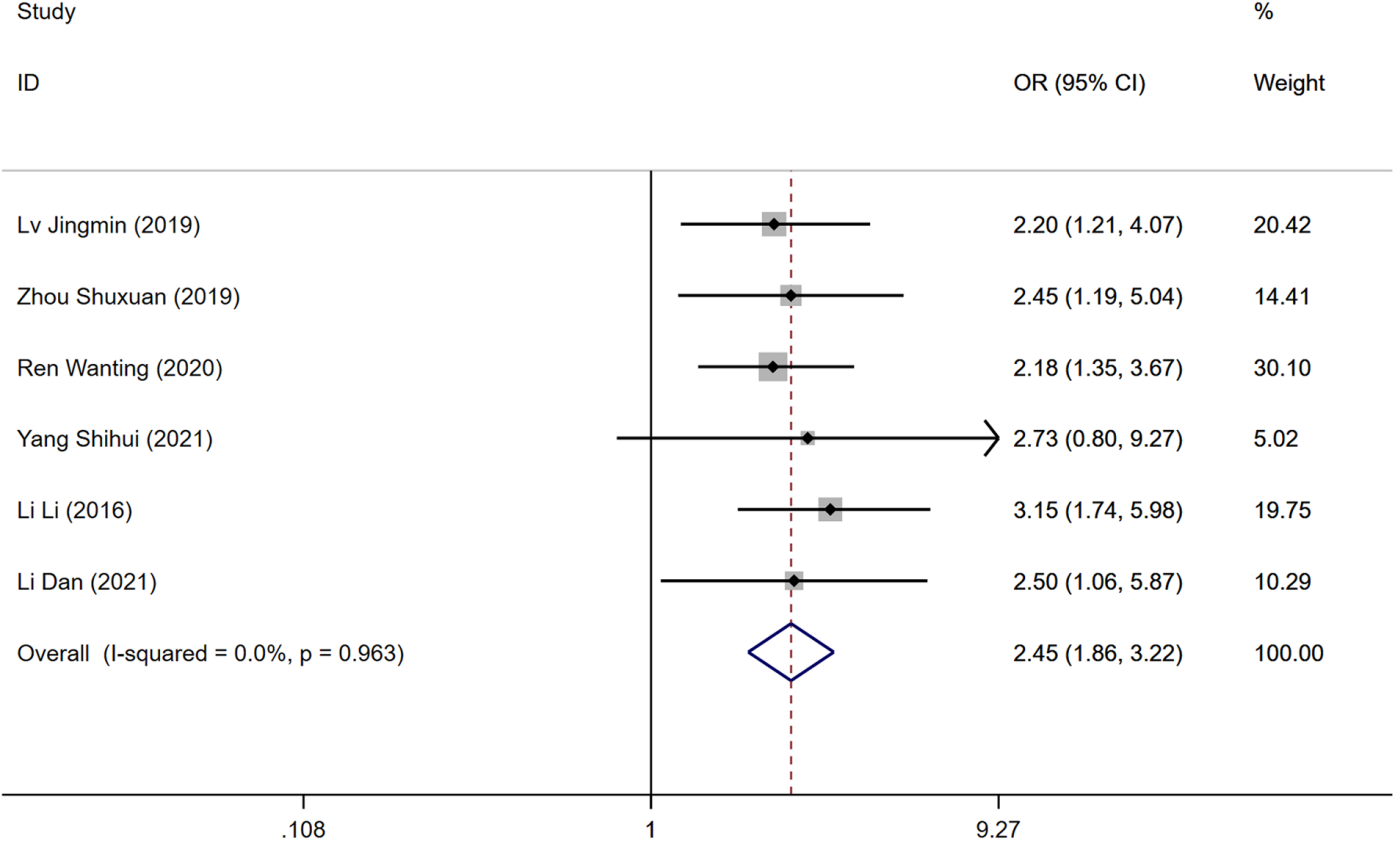
Forest plot of the relationship between malignancy and postoperative DVT in patients with gynecologic malignancies.

Six studies examined the presence of malignant tumors as a risk factor for DVT development post-surgery. The heterogeneity test indicated no significant heterogeneity (*P* = 0.963, *I*^2^ = 0.0%), allowing for the utilization of a fixed-effects model. The analysis revealed that while malignant tumors were considered a risk factor, there was no statistically significant difference in the incidence of DVT between the DVT group and the control group [OR = 2.45, 95% CI (1.86, 3.22), *P* = 0.963], as shown in Figure 4.

### Duration of surgery

**Figure 5:**
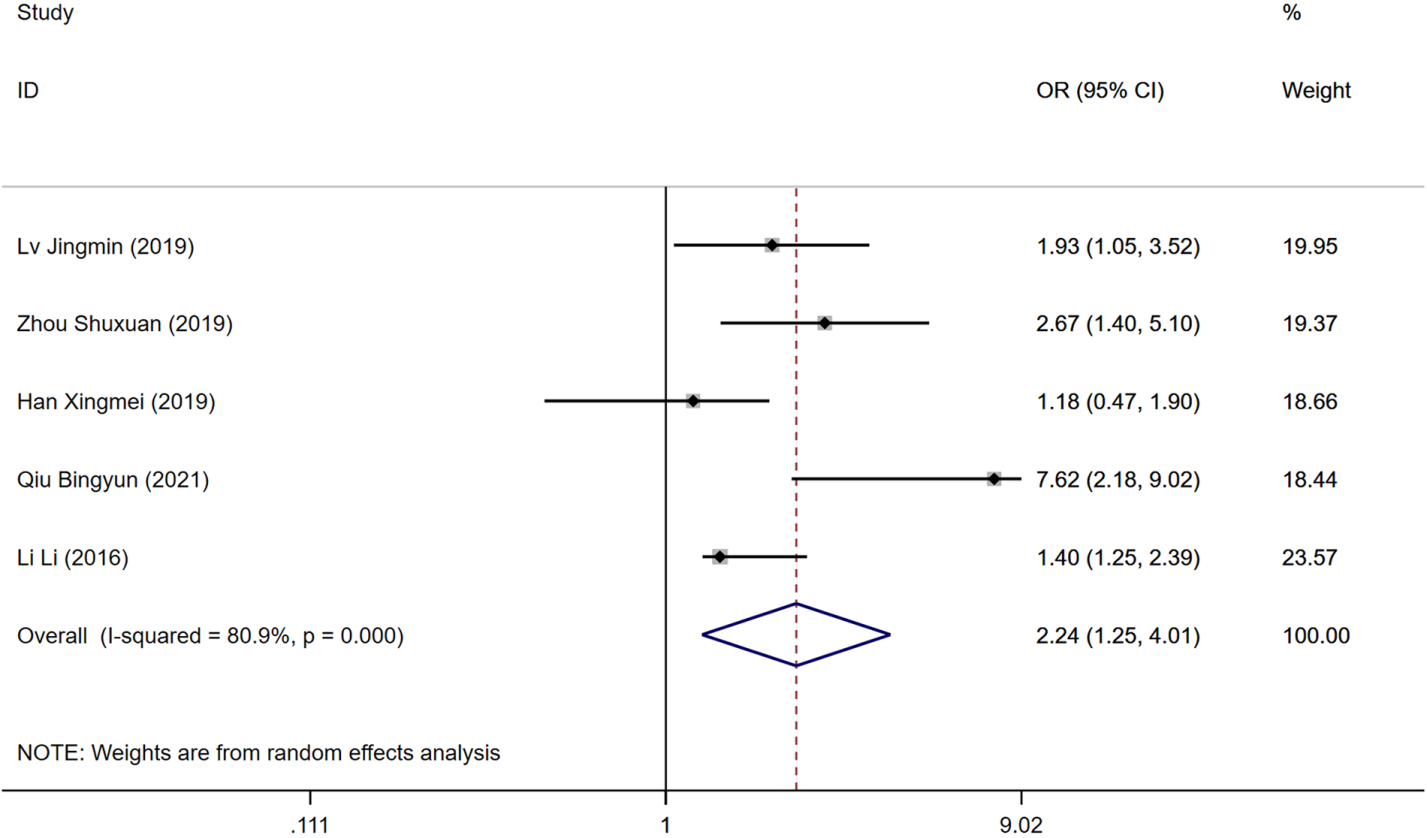
Forest plot of the relationship between operation time and postoperative DVT in patients with gynecologic malignancies.

The duration of the surgical procedure was reported as a risk factor in five studies. Significant heterogeneity was detected (*P* = 0.000, *I*^2^ = 80.9%), necessitating a random-effects model for analysis. Longer surgery durations were associated with a higher risk of developing DVT post-operatively. A statistically significant difference was found between the DVT and control groups [OR = 2.24, 95% CI (1.24, 4.01), *P* = 0.000], as illustrated in Figure 5.

### Postoperative bedtime

**Figure 6:**
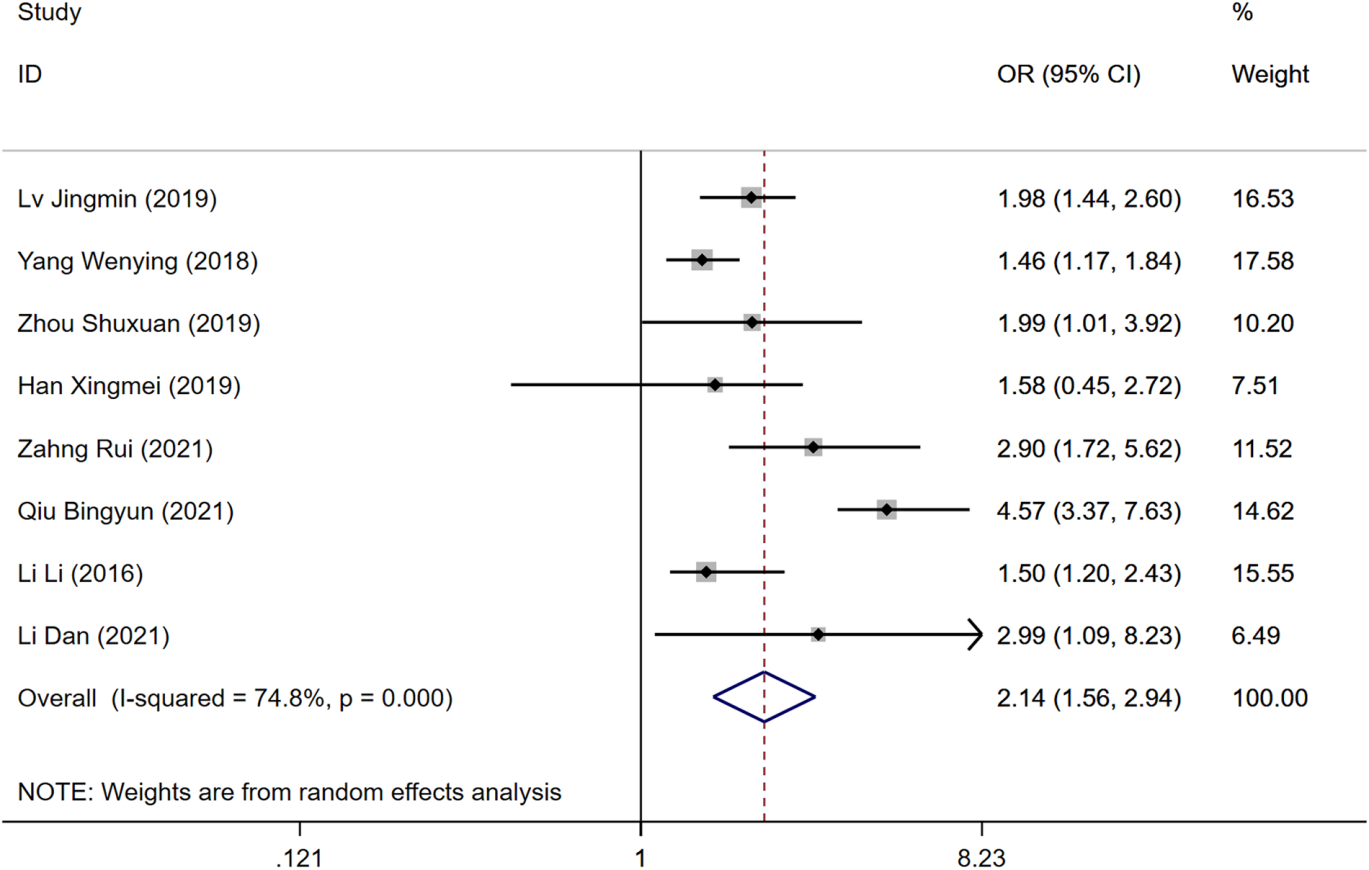
Forest plot of the relationship between postoperative bedtime and postoperative DVT in patients with gynecologic malignancy.

Eight studies explored the impact of postoperative bed rest duration as a risk factor for DVT development following gynecologic cancer surgery. Significant heterogeneity was observed (*P* = 0.000, *I*^2^ = 74.8%), analyzed with a random-effects model. Extended periods of bed rest post-surgery were identified as a risk factor for DVT, although the difference in DVT incidence between the DVT group and the control group was not statistically significant [OR = 2.14, 95% CI (1.56, 2.94), *P* = 0.000], as shown in Figure 6.

### Publication bias

**Figure 7:**
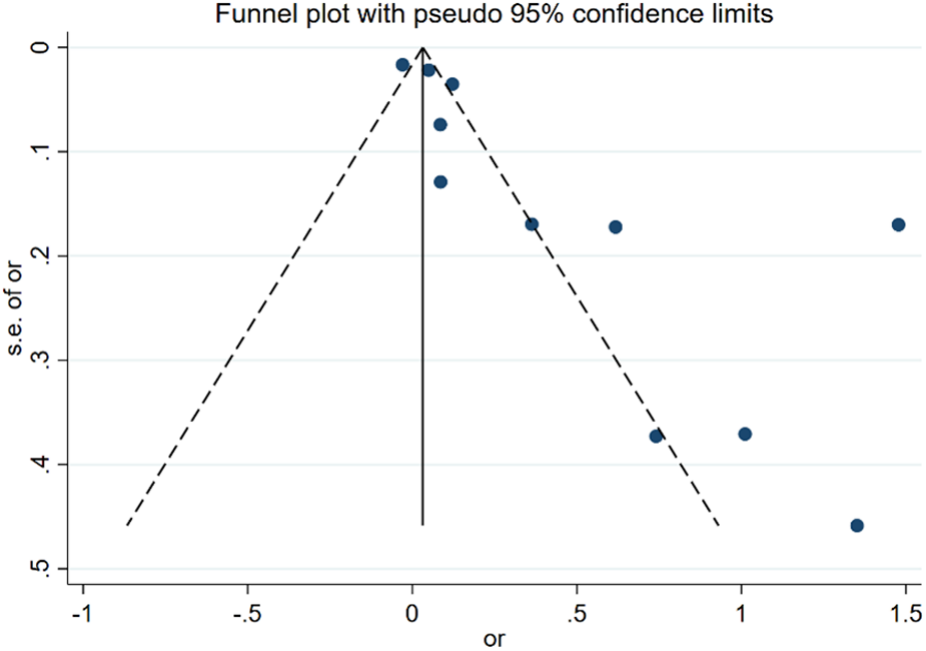
Funnel plot of the relationship between age and postoperative DVT in patients with gynecologic malignancies.

The assessment of publication bias focused on age as a risk factor for DVT in postoperative gynecologic malignancy patients. A funnel plot was constructed using the mean difference (MD) values from each study as the horizontal axis and the inverse of the logarithm of the standard error of the MD values as the vertical axis. The resulting asymmetry in the plot suggests the presence of publication bias among the included studies, as depicted in Figure 7.

## Discussion

Deep Vein Thrombosis (DVT) constitutes a pathological condition characterized by abnormal coagulation of blood within the veins, leading to venous obstruction and compromised venous return. This condition manifests through four primary symptoms: limb swelling, pain, an increase in local skin temperature, and superficial vein distension. DVT is noted for its significant morbidity and mortality rates, representing a prevalent and severe complication within the realm of gynecological oncology. The primary risk factors for DVT encompass surgical procedures, malignancy, and prolonged immobility, with gynecological surgeries posing a heightened risk due to specific anatomical considerations and distinctive characteristics (17), Consequently, the prompt and precise diagnosis, followed by standardized management of DVT by healthcare professionals, is imperative (18). Research indicates a marked difference in the incidence rates of postoperative DVT between patients with benign gynecological conditions (14%) and those afflicted by malignant tumors (38%). Furthermore, the mortality rate among patients diagnosed with DVT is reported to be up to sixfold higher than the baseline mortality rate (19). This evidence underscores the critical nature of DVT and highlights the necessity for its prevention and effective management in the clinical setting.

Virchow's triad posits that thrombosis is precipitated by three principal factors: endothelial injury, alterations in hemodynamics, and hypercoagulability of the blood. The abundance of pelvic blood vessels in women, coupled with the potential endothelial damage incurred during gynecological surgeries, sets the stage for vascular injury (20). This risk is further compounded by the prolonged bed rest post-surgery, leading to slowed blood flow and hemodynamic changes that favor the development of lower extremity deep vein thrombosis (DVT). Research has elucidated that the risk of lower extremity venous thrombosis in patients with malignant tumors is significantly elevated—ranging from two to seven times higher—compared to the general population (21).

Further studies affirm age as a critical risk factor for the formation of DVT, establishing a direct correlation between advancing age and the incidence of lower extremity DVT (22). Notably, DVT manifests with particular prevalence at three peak ages: 20–24, 45–59, and 70–74 years. The transition into the perimenopausal period, characterized by decreased hormone levels, reduced activity, and increased vascular fragility, escalates the risk of vascular injury and, subsequently, the incidence of DVT in older women (23). For each year of age, the risk of developing lower extremity venous thrombosis increases by a factor of 1.07 relative to the general populace (24). Obesity serves as both a predisposing and exacerbating factor for numerous health conditions (25). Studies have verified that individuals with a Body Mass Index (BMI) greater than 25 face a 1.3-fold increase in DVT risk compared to those of normal weight (26, 27). Experts contend that obesity fosters a pro-inflammatory state, wherein the resultant inflammation augments the production of pro-coagulant factors within the bloodstream, thereby facilitating the genesis and progression of DVT.

Plasma D-dimer serves as a highly sensitive biomarker for the prophylaxis of deep vein thrombosis (DVT) in the lower extremities. Nevertheless, its utility is frequently limited in clinical practice due to the fact that elevated D-dimer levels can result from a variety of conditions, including but not limited to, hepatic and renal dysfunctions, as well as atrial fibrillation. Furthermore, patients with gynecologic malignancies who experience prolonged durations of surgery and postoperative bed rest constitute additional risk factors for the development of DVT. This association is particularly significant given the direct correlation with the impairment of lower limb venous return. Specifically, the extended operative times exacerbate damage to patient's tissues and organs, thereby eliciting a more rapid and pronounced systemic stress response. Consequently, the incidence of venous thrombosis in the lower limbs is quadrupled in patients whose surgeries exceed one hour in duration, compared to those with gynecologic oncology procedures under one hour (28). Additionally, the presence of malignant tumors markedly elevates the risk of DVT, with the extent of thrombotic risk being intricately linked to both the type and stage of the tumor (29). A relevant study suggests that low molecular weight heparin (LMWH) and fondaparinux have demonstrated greater efficacy in preventing DVT in gynecologic cancer patients, and that direct oral anticoagulants (DOACs) can be used to prevent DVT in outpatients and high-risk medical patients post-discharge (30).

In this research, we conducted a comprehensive analysis of the contributory factors to the incidence of Deep Vein Thrombosis (DVT) post-gynecologic oncology surgeries, synthesizing data from twelve studies. This analysis encompassed six key risk factors, revealing that significant predictors for postoperative DVT in patients with gynecologic malignancies include age, Body Mass Index (BMI), D-dimer levels, surgery duration, and the length of postoperative bed rest. Furthermore, the included literature indicates that the occurrence of DVT in the lower extremities is predominantly concentrated within the first 1–10 days post-surgery.

This study represents an extensive and generalized examination, building upon prior analyses that considered multiple factors in isolation. It highlights the multifaceted nature of risk factors associated with postoperative DVT in patients undergoing treatment for gynecologic malignancies. Furthermore, it addresses the current clinical debates surrounding these risk factors, selecting contentious issues as focal points for investigation. Nonetheless, this study is not without its limitations. This study lacks the inclusion of relevant clinical parameters, as the original authors of the included literature did not consistently report specific cutoff values for variables such as age, body mass index, D-dimer levels, duration of surgery, and length of postoperative bed rest. Additionally, the available data in various studies were not uniformly categorized, and no clear threshold for the analysis of continuous variables was established, which may affect the validity of the study's results. The study exclusively relies on published literature, which may lead to an insufficient number of studies and sample sizes, coupled with an inherent risk of bias due to the retrospective nature of these studies. Moreover, the literature review was limited to Chinese publications, potentially omitting relevant studies and thereby constraining the comprehensiveness of the analysis. The risk of bias assessment for the included studies indicated a high risk of bias. Notably, the selected literature did not account for patients who experienced thrombus resolution in the preoperative period, introducing a potential selection bias. Consequently, there is a pressing need for corroborative studies with larger sample sizes to establish a more robust evidence base for clinical decision-making in the context of evidence-based medicine.

## Data Availability

The original contributions presented in the study are included in the article/Supplementary Material, further inquiries can be directed to the corresponding author.
